# The diversity of the Baikal lineage of *Hydra oligactis* Pallas, 1766: molecular and morphological evidence

**DOI:** 10.3897/zookeys.912.46898

**Published:** 2020-02-17

**Authors:** Tatiana E. Peretolchina, Igor V. Khanaev, Ilya V. Enushchenko, Dmitry Y. Sherbakov, Lyubov S. Kravtsova

**Affiliations:** 1 Limnological Institute SB RAS, Ulan-Batorskaya Str., 3, 664033, Irkutsk, Russia Limnological Institute SB RAS Irkutsk Russia

**Keywords:** taxonomy, holotrichous isorhizas, phylogeny, Baikal hydras

## Abstract

In this paper, molecular analyses of Baikal hydras from the ‘*oligactis* group’, based on COI and ITS1–5.8S–ITS2, and morphological analysis of their holotrichous isorhizas, were performed. Low genetic diversity and shared haplotypes were found between *Hydra oligactis* Pallas, 1766 and *Hydra baikalensis* Swarczewsky, 1923 specimens, which is evidence of the mixing of these lineages. Genetic distances among all Baikal hydras (0.006) were less than the interspecific distances of other hydras. The size of hydras and proportions of their holotrichous isorhizas varied depending on microhabitat and environmental conditions. Our combined molecular and morphological approach proves that *H. baikalensis* is synonymous with *H. oligactis*

## Introduction

*Hydra* is a member of the ancient phylum Cnidaria, class Hydrozoa, order Hydroida, family Hydridae. Freshwater representatives of *Hydra* inhabit virtually all zoogeographical regions except the Pacific Ocean Islands and Antarctica (Jankowski et al. 2008), living in lenthic and lothic waters such as ponds, lakes, and rivers, and prefering meso-eutrophic conditions. Lake Baikal is one of the deepest (1642 m) oligotrophic freshwater lakes in the world, but the habitat conditions in its bays are close to meso-eutrophic. Climate warming is currently considered as a key factor capable of stimulating the eutrophication of freshwater ecosystems (Cohen et al. 2016; Paerl and Otten 2013). According to the World Meteorological Organization, 2010 was one of the hottest years in the history of meteorological observations. Global climate changes are affecting the ecosystem of Lake Baikal as well. Structural rearrangements of the phytoplankton community in the pelagic zone of the lake (Bondarenko et al. 2019) and changes in the structure of bottom phytocenoses of the coastal zone (Kravtsova et al. 2014) have been detected. Moreover, the propagation of Palearctic species of invertebrates such as *Lymnaea* mollusks (Gastropoda, Lymnaeidae) and the caddis fly *Apatania majuscula* McLachlan, 1872 (Insecta, Trichoptera, Apataniidae) have been observed in Lake Baikal (Rozhkova et al. 2018). In addition, the mass death of the endemic species of Porifera sponges (Denikina et al. 2016; Khanaev et al. 2018) has also been recently observed. On the contrary, representatives of *Hydra* have begun to play a significant role along the open coasts of Lake Baikal. Previously, representatives of hydras were found in small numbers in the coastal zone of Lake Baikal. The first record of the mass development of *Hydra oligactis* Pallas, 1766 was in the 2000s (Stepanyants et al. 2003). Now, *Hydra* is abundant along the open coasts of all three basins of Lake Baikal (Peretolchina et al. 2018a).

Of the 80 *Hydra* species names described in the world only 12 to 15 are valid (Jankowski et al. 2008). The uncertainty of the position of individual hydra taxa results from the complexity of their identification, due to their primitive structure and limited number of reliable diagnostic features. The species diversity of *Hydra* within the morphological boundaries of Lake Baikal is not high; there are four species of *Hydra* reported from the Baikal Region: *H. oligactis* Pallas, 1766 and *Hydra baikalensis*Swarczewsky 1923, as well as the rare *Hydra circumcincta* Schulze, 1914 and *Hydra oxycnida* Schulze, 1914 (Peretolchina et al. 2018b). *Hydra baikalensis*, *H. oligactis* and *H. oxycnida* belong to the ‘*oligactis* group’ (Hemmrich et al. 2007; Martínez et al. 2010; Schwentner and Bosh 2015). However, the morphological descriptions of *H. baikalensis* and *H. oligactis* are very similar in both polyp morphology and holotrichous isorhiza structure (Swarczewsky 1923; Anokhin 2002).

The aim of our study was to verify the identity of *H. oligactis* and *H. baikalensis* using both morphological and molecular data.

## Materials and methods

Hydras were collected by scuba divers along southwestern littoral areas (Bolshie Koty – 51°54.04'N, 105°04.08'E, Sobolev Cape – 51°54.20'N, 105°10.18'E, Listvennichniy Bay – 51°51.24'N, 104°51.61'E) of Lake Baikal at depths of 5 to 18 meters, together with dying Porifera and *Nitella* algae, and by hand from the eastern shores (Posolskiy Sor – 51°57.21'N, 106°05.65'E, Barguzinskiy Bay – 53°16.65'N, 108°44.01'E, Chivyrkuiskiy Bay – 53°38.43'N, 109°04.03'E) of Lake Baikal at a depth of 1 meter, together with the aquatic plants *Potamogeton perfoliatus* Linnaeus, 1753 and *Potamogeton lucens* Linnaeus, 1753. Typically, representatives of *Hydra* were brought live into the laboratory, but some samples were fixed in 80% ethanol in the field.

Morphological studies of the cnidome (stenoteles, desmonemes, holotrichous and atrichous isorhizas) were carried out using an Olympus CX22 microscope with 1000-fold magnification under oil immersion. Morphometric measurements of holotrichous isorhizas were made with the Image-Pro program. *Hydra* specimens were identified according to keys provided by Schuchert (2010, 2018) and Anokhin (2002).

DNA was extracted from a single live or fixed individual as described by Doyle and Dickson (1987). Gene fragments of mitochondrial cytochrome c oxidase subunit I (COI) were amplified using standard Folmer primers for invertebrates (Folmer et al. 1994), and the internal transcribed spacer 1, 5.8S ribosomal DNA and internal transcribed spacer 2 (ITS1–5.8S–ITS2) were amplified using primers published in White et al. (1990).

All PCR reactions were performed in a final volume of 15 μL using 2-Red PCR mix (10 X PCR buffer, 50 mM MgCI2 and 0.02 unit/ μL Taq DNA polymerase). The PCR amplification conditions were as follows: denaturation at 94 °C for 5 min, 30 cycles at 94 °C for 30 sec, 50 °C for 45 sec, 72 °C for 2 min, and a final elongation step at 72 °C for 10 min. Direct sequencing of forward sequences was performed using an ABI 3130 automated sequencer (Research and Production Company “SYNTOL”, Moscow, Russia).

The DNA sequences obtained were aligned using default settings in CLUSTAL W (Thompson et al. 1994), implemented in BIOEDIT v.7.2.5 (Hall 1999) and optimized by eye. The resulting COI alignment was translated to check for the absence of stop codons. To confirm species identity, we compared our sequence dataset to published orthologous sequences from other members of *Hydra* and analyzed genetic distances using MEGA v.6 (Tamura et al. 2013).

Phylogenetic analyses were performed using MRBAYES v.3.2 (Ronquist and Huelsenbeck 2003). The datasets for the COI and ITS1–5.8S–ITS2 fragments (Table 1) consisted of unique haplotypes of Baikal hydras produced for this study and specimens (three species from the ‘*oligactis* group’: *Hydra canadensis* Rowan, 1930, *H. oligactis*, *H. oxycnida*, and a representative of *H. circumcincta*, as outgroup) retrieved from GenBank. To estimate the posterior probabilities of the phylogenetic tree, we used 20,000,000 generations of Metropolis-coupled Markov chain Monte Carlo simulation (two runs with four chains). We used the JMODELTEST v.2.1 (Darriba et al. 2012) to determine the substitution models for the two genes separately. The best-fit model for phylogenetic analysis in the case of COI was HKY, and in the case of ITS1–5.8S–ITS2 it was GTR+I+G. We constructed a majority-rule (50%) consensus tree following 25% burn-in of all sampled trees.

**Table 1.** List of used specimens with accession numbers provided.

**Species name**	**GeneBank accession numbers**	**ITS1–5.8S–ITS2 (reference)**
	**COI (reference)**	
*H. oligactis*	GU722865–GU722875 (Martínez et al. 2010);	GU722678–GU722688 (Martínez et al. 2010)
	AB565122, AB565130 (Kawaida et al. 2010);	
	KP895118 (Schwentner and Bosch 2015);	
	MF000491, MF544747, MF135286, MF135293, MF135295, MF135298, MF135299, MF135302, MF135304, MF135305, MF13531 (Schuchert, unpublished)	
*H. robusta*	EF059939 (Hemmrich et al. 2007);	
	KP895119 (Schwentner and Bosch 2015);	
	HQ417108 (Wang et al. 2012);	
	AB565143, AB565125, AB565094, AB565093, AB565092 (Kawaida et al. 2010)	
*H. oxycnida*	GU722876, GU722877 (Martínez et al. 2010);	GU722689 (Martínez et al. 2010)
	KP895120 (Schwentner and Bosch 2015)	
*H. canadensis*	GU722879–GU722884 (Martínez et al. 2010)	GU722697 (Martínez et al. 2010)
Baikal *H. oligactis*	MH428229–MH428232, MH428234–MH428235, MH428269–MH428270, MH428273, MH428275– MH428276, MH428278– MH428279, MH428281, MH428288, MH428292, MH428294, MH428304, MH428318, MH428338, MH428341, MH428343, MH428347, MH428349–MH428350, MH428354, MH428358–MH428360, MH428363 (Our data)	MH454379, MH454381, MH454383–MH454386, MH454390, MH454393, MH454398, MH454403, MH454407–MH454408, MH454412, MH454419, MH454427–MH454433, MH454437, MH454439, MH454441, MH454443–MH454444, MH454446, MH454450–MH454452, MH454455 (Our data)

## Results

### Molecular analysis

In total, we produced 30 COI sequences (603 bp long) and 30 ITS1–5.8S–ITS2 sequences (up to 710 bp long). Inspection of the Baikal hydra sequences revealed nine unique haplotypes for the COI gene fragment (shared haplotypes were found in all sampling localities) and three unique haplotypes for the ITS1–5.8S–ITS2 sequences.

**Figure 1.**
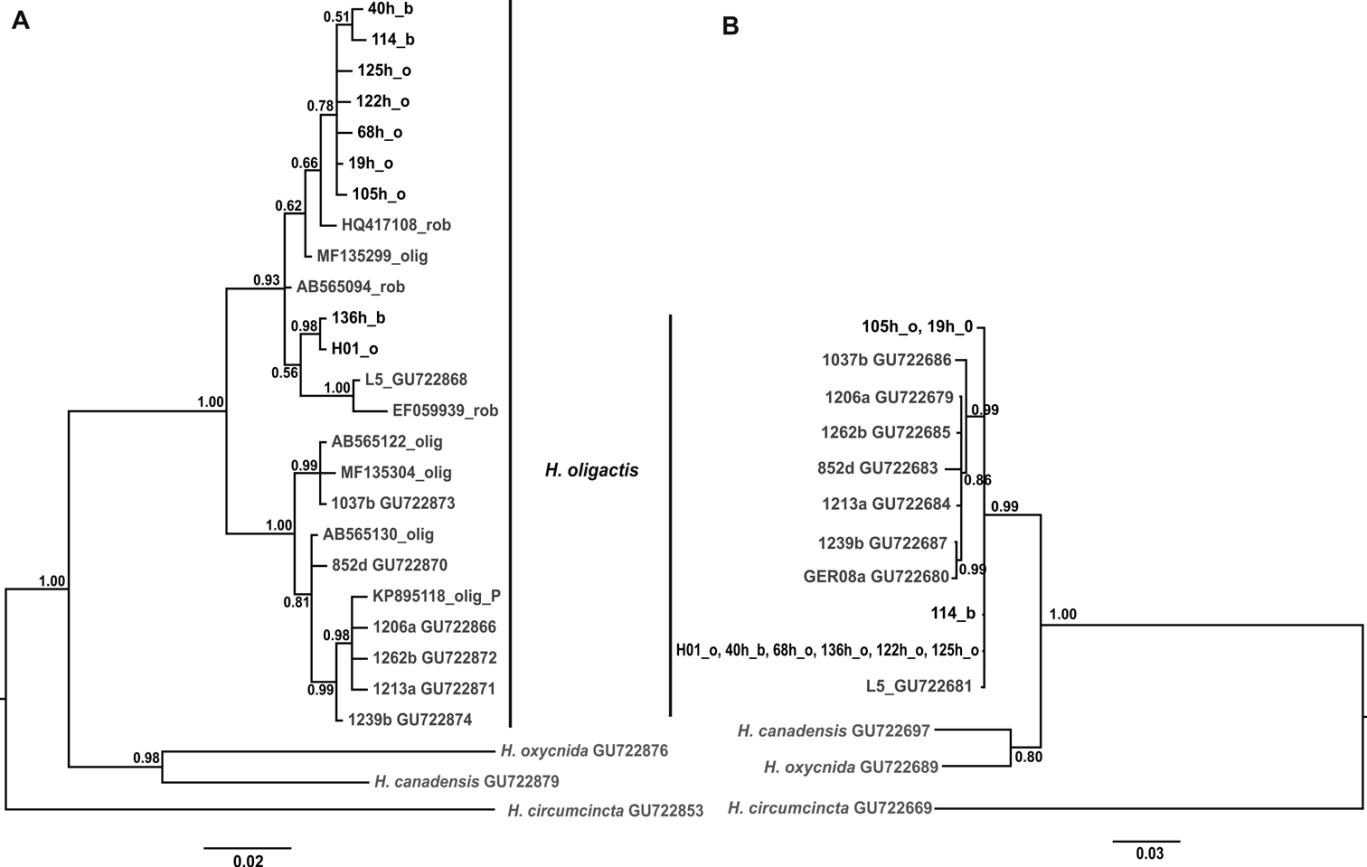
Bayesian phylogenetic tree based on COI (**A**) and ITS1–5.8S–ITS2 unique haplotype sequences. Posterior probabilities (>0.5) are given at nodes. Tip names with ‘_o’ belong to *H. oligactis* specimens and those with ‘_b’ belong to *H. baikalensis* specimens. Tip names marked in grey color indicate nucleotide sequences from GenBank.

The consensus tree topology based on COI (27 unique haplotypes) indicated that Baikal *H. oligactis* and *H. baikalensis* did not form separate clades, but instead were clustered together with representatives of *Hydra robusta* Itô, 1947 from Japan and China and *H. oligactis* from Japan and Europe, forming a neighboring clade with the majority of hydras from Western Europe and North America (Fig. 1A). The consensus phylogenetic tree based on ITS1–5.8S–ITS2 (14 unique haplotypes) showed that differentiation between hydras from Europe + North America and Asia was not evident (Fig. 1B).

Mean pairwise *p*-distances based on COI sequences of *Hydra* species are given in Table 2. Intraspecific genetic distances of *Hydra* varied from 0.010 to 0.040, whereas interspecific distances were about 0.100. The mean genetic distance between Baikal hydras and *H. oligactis* from Western Europe and North America was 0.030 (min 0.009, max 0.035). The mean *p*-distance for lineages of *H. oligactis*, Baikal hydras and *H. robusta* was 0.024, which corresponds to the intraspecific variability in *Hydra* species (Table 2).

**Table 2.** Pairwise p-distances between COI sequences of different species of the genus Hydra. Within group mean pairwise distances are in bold.

	**1**	**2**	**3**	**4**	**5**
**1 – *H. oligactis* (Baikal)**	**0.006**				
**2 – *H. oligactis***	0.030	**0.018**			
**3 – *H. robusta***	0.017	0.035	**0.017**		
**4 – *H. oxycnida***	0.103	0.101	0.103	**0.040**	
**5 – *H. canadensis***	0.101	0.103	0.105	0.105	**0.010**

### Morphological analysis

In our collections, hydras with a length/width ratio (L:W) of holotrichous isorizas > 2 were identified as *H. oligactis* and hydras with L:W ≤ 2 were identified as *H. baikalensis*, following Schuchert (2010, 2018) and Anokhin (2002). However, morphological analysis of polyps did not reveal specific features between Baikal hydras and *H. oligactis*, and the main diagnostic trait (L:W of holotrichous isorizas) varied from 1.85 to 2.46 (Table 3), depending on the environment, in different sites of Lake Baikal. Hydra specimens with a L:W of holotrichous isorizas ≤ 2.0 were collected at a depth of 15–18 m, where the water temperature was low (6–7 °C, near the bottom). Hydra specimens with holotrichous isorizas ≥ 2.0 were collected in shallow water from 1–5 m, where the water temperature reaches 16–24 °C in summer (Kozhov 1962).

**Table 3.** Holotrichous isorhizas measuring.

**Site**	**Length**, μm	**Width**, μm	**L:W**	**n**
Chivirkuiskiy Bay	9.46±0.17	3.90±0.07	2.44±0.06	17
Barguzinskiy Bay	9.35±0.15	3.81±0.06	2.46±0.04	17
Lystvenichniy	8.26±0.18	4.04±0.08	2.06±0.06	20
Bolshie Koty	8.42±0.10	4.24±0.09	2.02±0.04	42
Sobolev	7.74±0.06	4.19±0.07	1.85±0.03	11
Posolskiy Sor	10.04±0.19	4.24±0.08	2.38±0.05	27

## Discussion

According to Schwentner and Bosch (2015), the ‘*oligactis* group’ includes *H. oxycnida*, *H. canadensis*, *H. oligactis* and *H. robusta* (possibly synonymous to *H. oligactis*). On the one hand, low genetic distance between the latter two species could be a consequence of shared ancestral polymorphism. On the other hand, the results may be due to population genetic variability of *H. oligactis* (= *H. robusta*). It is known that genetic polymorphism of species with wide geographic distributions is higher than that of species with restricted ranges, where genetic diversity decreases from the center of distribution to range boundaries (Brown 1984; Schuchert 2014). We suppose that *H. oligactis* is a widely distributed species with genetic (population) structure (Figure 1A, B). The “Baikal” clade includes not only regional haplotypes but also haplotypes of *H. oligactis* from Europe, China and Japan (Fig. 1A). We believe that this level of genetic diversity corresponds to population differences, not to interspecific ones. Thus, according to our molecular phylogenetic study, all the haplotypes belong to the same species, *H. oligactis* (*H. oligactis* = *H. robusta* = *H. baikalensis*).

In Lake Baikal, the type locality of the endemic *H. baikalensis* is Chivyrkuyskiy Bay (Swarczewsky 1923). This species has also been reported from Listvennichniy Bay, the littoral zone near Bolshoy Ushkaniy Island, Bolshie Koty Bay, and in small lakes along the Bolshie Koty River. *Hydra oligactis* occurs in the same areas and was also reported from Dagarskaya Bay, Mukhor Bay (Stepanyants et al. 2006), and Barguzinskiy Bay (Peretolchina et al. 2018a).

Previous researchers (Schulze 1927; Anokhin 2002; Jankowski and Anokhin 2019) distinguished *H. oligactis* from *H. baikalensis* mostly based on differences in the proportions of their holotrichous isorhizas. However, the differences in the length/width ratio of holotrichous isorhizas in *H. baikalensis* and *H. oligactis* are within the range of intraspecific variability (Table 3). Therefore, this morphological feature cannot be used as a diagnostic criterion for *H. baikalensis*. It is known that proportions and relative sizes of the nematocysts vary considerably depending on the stage of development of the nematocysts (Weill 1934) and on environmental factors (Östman 1987; Itô 1951). In our case, the size of the hydras and the proportions of the holotrichous isorhizas varied depending on microhabitat and environmental conditions. That is, specimens sampled in the open littoral of Lake Baikal were larger and their holotrichous isorhizas were shorter and slightly thicker (8–9 μm, length/width ratio ≤ 2), whereas hydras from shallow depths were smaller but their holotrichous isorhizas were longer and thinner (9–10 μm, length/width ratio > 2) (Table 3). In addition, the packing of stinging threads in the holotrichous isorhizas of both Baikal *H. baikalensis* and *H. oligactis* was the same.

Moreover, the species *H. baikalensis* and *H. oligactis* are similar in the number and morphology of chromosomes, in the symmetrical structure of the karyotype, and in C-heterochromatin localization. They only differ in the length ratio of the 1^st^ and 15^th^ pairs of chromosomes (Anokhin 2002). We suppose that the length ratio of chromosomes is a distinctive feature but may be not sufficient to distinguish *H. baikalensis* as an independent species.

In addition, molecular phylogenetic analyses did not reveal any differences between *H. baikalensis* and *H. oligactis* specimens. Genetic distances among all Baikal hydras (0.006) were less than the interspecific distances of other hydra (Table 2). Moreover, we found shared haplotypes among representatives of Baikal *H. baikalensis* and *H. oligactis*. The sympatric occurrence of *H. oligactis* and *H. baikalensis* and the low level of their genetic distances (revealed using both mitochondrial and nuclear markers) do not allow us to consider these two groups as separate species.

Thus, the morphological characteristics, patterns of haplotype diversity, and the results of phylogenetic analyses lead us to consider *H. baikalensis* as a synonym of *H. oligactis*.

## Systematics

Phylum Cnidaria Verrill, 1865

Subphylum Medusozoa Peterson, 1979

Class Hydrozoa Owen, 1843

Subclass Hydroidolina Collins 2000

Order Anthoathecata Cornelius, 1992

Family Hydridae Dana, 1846

*Hydra oligactis* Pallas, 1766

*Hydra fusca* Linnaeus, 1767

*Hydra roeselii* Haacke, 1879

*Hydra rhaetica* Asper, 1879

*Hydra rhistica* Asper, 1880

? *Hydra monoecia* Downing, 1900

? *Hydra pallida* Beardsley, 1904

? *Hydra corala* Elrod & Ricker, 1902

? *Hydra dioecia* Downing, 1905

*Pelmatohydra oligactis* (Pallas, 1776)

*Hydra baikalensis* Swarczewsky, 1923

**Figure 2.**
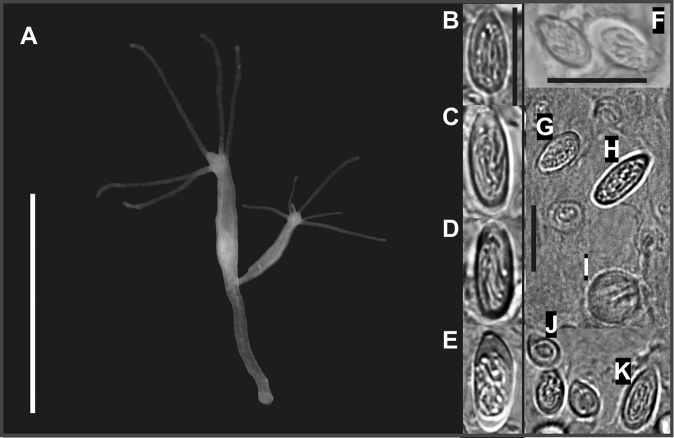
**A***H. oligactis*, living polyp from Lake Baikal. **B**–**F** morphological variability of holotrichous isorhizas **B**, **F***H. baikalensis*; **C**, **D**, **E***H. oligactis* (**B** Bolshie Koty **C** Posolskiy Sor **D** Chivyrkuiskiy Bay **F** Sobolev) **E** Photo adapted from Schuchert (2010). **J** desmonemes **F**, **G** atrichous isorhizas **H**, **K** holotrichous isorhizas **I** stenoteles. Scale bars: 5 mm (**A**), 10 μm (**B**–**K**).

**Figure 3.**
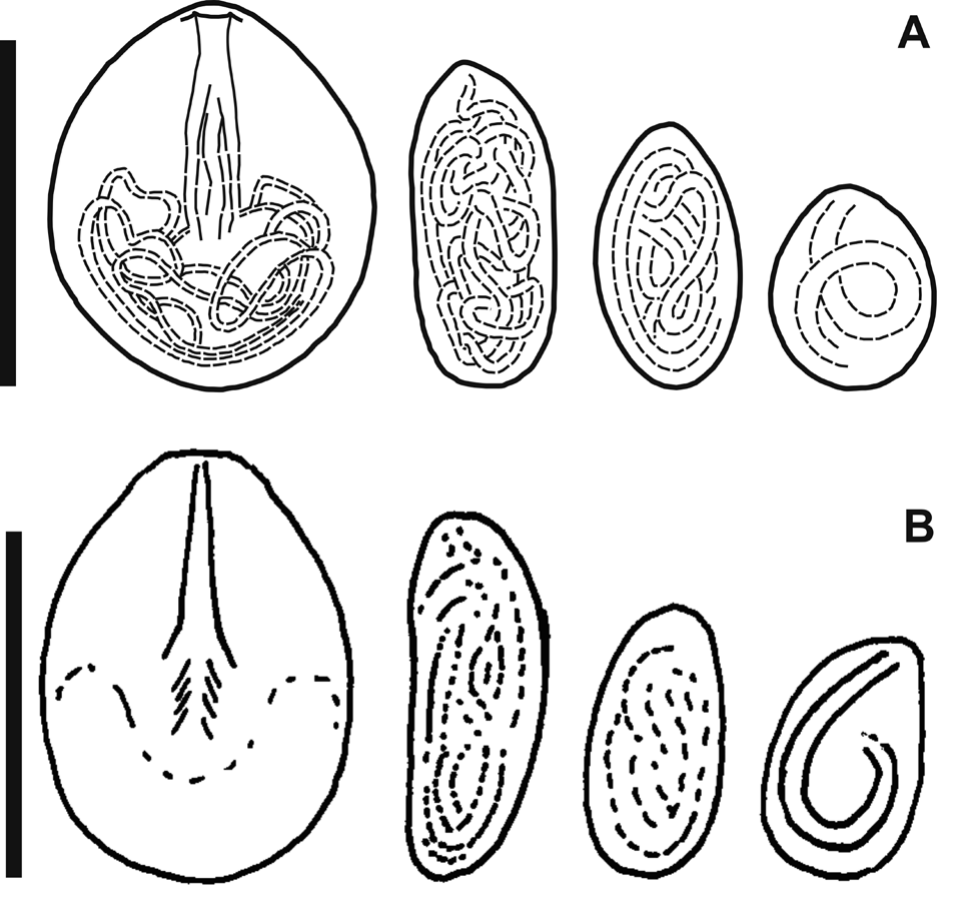
**A** cnidome of Baikal *H. oligactis***B** cnidome of *H. oligactis*, figure adapted from Schuchert (2010). From left to right: stenoteles, holotrichous isorhizas, atrichous isorhizas and desmonemes. Scale bars: 10 μm.

**Diagnosis.** The Baikal *Hydra* is large, typically with 5–7 long tentacles, and a more or less distinct peduncle. The cnidome includes four types of nematocysts: stenoteles, holotrichous isorhizas, atrichous isorhizas and desmonemes (Figs 2, 3). Holotrichous isorhizas are elongated, with a length/width ratio of about 2, and with thread-forming longitudinal, irregular coils inside the capsule. Other types of nematocysts are of a size and shape typical of other hydras. Holotrichous isorhiza measurements are summarized in Table 3, with photographs in Fig. 2.

**Distribution.** Baikal region: Port Baikal, Ulanovo, Listvennichny Bay, Bolshie Koty, Varnachka, Sobolev Cape, Turali Cape, Elokhin Cape, Mukhor Bay, Posolsky Sor Dagarskaya Bay, littoral zone near Bolshoy Ushkaniy Island, small lakes along the Bolshie Koty River, and Lake Kuzmikhinskoye (Artificial reservoir near the Angara River). This species is also widespread and common on the entire European continent, including the British Isles and Iceland as well as Russia and North America (Hyman 1930; Holstein 1995; Stepanyants et al. 2006).
